# Patient Transfers and Risk of Back Injury: Protocol for a Prospective Cohort Study With Technical Measurements of Exposure

**DOI:** 10.2196/resprot.8390

**Published:** 2017-11-08

**Authors:** Jonas Vinstrup, Pascal Madeleine, Markus Due Jakobsen, Kenneth Jay, Lars Louis Andersen

**Affiliations:** ^1^ Department of Musculoskeletal Disorders National Research Centre for the Working Environment Copenhagen Denmark; ^2^ Department of Health Science and Technology Physical Activity and Human Performance Group, SMI Aalborg University Aalborg Denmark; ^3^ The Carrick Institute for Graduate Studies Institute of Clinical Neuroscience and Rehabilitation Florida, FL United States

**Keywords:** nurse, low back pain, electromyography, assistive devices, pain, hospital

## Abstract

**Background:**

More than one third of nurses experience musculoskeletal pain several times during a normal work week. Consistent use of assistive devices during patient transfers is associated with a lower risk of occupational back injuries and low back pain (LBP). While uncertainties exist regarding which type of assistive devices most efficiently prevent LBP, exposure assessments using technological advancements allow for quantification of muscle load and body positions during common work tasks.

**Objective:**

The main objectives of this study are (1) to quantify low back and neck/shoulder muscle load in Danish nurses during patient transfers performed with different types of assistive devices, and (2) to combine the exposure profile for each type of assistive device with fortnightly questionnaires to identify the importance of muscle load (intensity and frequency of transfers) and body position (degree of back inclination and frequency) on LBP intensity and risk of back injury during a patient transfer.

**Methods:**

A combination of technical measurements (n=50) and a prospective study design (n=2000) will be applied on a cohort of female nurses in Danish hospitals. The technical measurements will be comprised of surface electromyography and accelerometers, with the aim of quantifying muscle load and body positions during various patient transfers, including different types of assistive devices throughout a workday. The study will thereby gather measurements during real-life working conditions. The prospective cohort study will consist of questionnaires at baseline and 1-year follow-up, as well as follow-up via email every other week for one year on questions regarding the frequency of patient transfers, use of assistive devices, intensity of LBP, and back injuries related to patient transfers. The objective measurements on muscle load and body positions during patient handlings will be applied to the fortnightly replies regarding frequency of patient transfer and use of different assistive devices, in order to identify risk factors for back injuries related to patient transfers and intensity of LBP.

**Results:**

Data collection is scheduled to commence during the winter of 2017.

**Conclusions:**

The design of this study is novel in its combination of technical measurements applied on a prospective cohort, and the results will provide important information about which assistive devices are associated with intensity of LBP and risk of back injury related to patient transfers. Furthermore, this study will shed light on the dose-response relationship between intensity, duration, and frequency of patient transfers and the intensity of LPB in Danish nurses, and will thereby help to guide and improve electronic health practices among this population.

## Introduction

Musculoskeletal disorders (MSDs) represent a widespread occupational disease as well as a major socioeconomic burden on public health systems in Europe and North America [1,2]. Low back pain (LBP) and neck/shoulder disorders are the main culprits of a physically demanding job, often leading to increased sickness absence and loss of productivity [2-4]. A number of work-related risk factors for developing MSDs have been highlighted in the literature. For example, heavy lifting as well as frequent bending and twisting of the spine have repeatedly been associated with increased risk of developing LBP [2,5,6]. Additionally, a meta-analysis from 2014 showed that both intensity and frequency of lifting tasks predict the occurrence of LBP [7]. Therefore, even though the development of MSDs has a complex etiology comprised of individual physical and psychosocial factors, it is clear that certain occupations and job tasks pose an inherent risk of developing MSDs.

One occupation that is commonly associated with MSDs as a result of physically demanding job tasks is the health care profession. The annual prevalence of MSDs among nurses and nurses’ aides is high (ie, 55% for LBP [8]). Furthermore, more than 36% of Danish nurses experience musculoskeletal pain several times during a normal work week [9]. These numbers not only reflect serious and broad-ranging health issues for the nurses in question, but will also have the potential to pose significant negative consequences for their patients. For example, as an inherent component of persistent pain, it is likely that nurses will decrease the number and quality of patient handlings (with and without assistive devices) due to LBP, which effectively diminishes the services of the hospital.

It is well-known that the utilization of various assistive devices during patient transfers decreases the risk of back injuries and intensity of LBP [10,11]. Furthermore, both ergonomic interventions [12] and the application of a “no lifting policy” [13] have proven effective in reducing work-related back injuries in hospitals. Despite these developments, most health care personnel report that they do not use appropriate assistive devices when moving patients [14]. A recent Danish cohort study showed that staff members performing daily patient transfers were more prone to back injuries one year later (odds ratio [OR] 1.56-1.81) compared with their counterparts who did not move patients on a daily basis [10]. An equally important finding from this cohort study was that the staff members who used assistive devices during patient transfers experienced a markedly lower risk of back injuries related to patient transfers one year later (OR 0.59-0.62) [10]. However, despite the clear indications that utilizing appropriate assistive devices during patient transfers in hospitals has a positive effect on the number of back injuries and prevalence of LBP, it is still unknown which assistive devices are most effective in terms of LBP prevention.

Previous research has used biomechanical measurements to identify peak loading during various job-related tasks among health care workers [15-19]. For example, muscular load has been shown to be significantly lower during patient transfers when using a ceiling-attached lift compared to traditional manual lifts from the floor [18]. Following this study, laboratory research by Schibye et al [20] illustrated the effect of lifting technique, showing that a self-chosen technique results in higher levels of spinal loading compared with the recommended patient transfer technique. This finding was further confirmed by another laboratory study showing that low back compression forces were influenced more by the health care worker’s technique and use of appropriate assistive devices than the weight and disability of the patient being repositioned in bed [21]. From the literature cited above, it appears that the appropriate use of assistive devices is of great importance when the goal is to decrease the prevalence of MSDs and the accompanying high amount of sickness absence in this population of nurses. However, only a small number of studies have measured work-related muscular load during a full real-life work day [22-24], and no study has investigated this among health care personnel.

The primary aim of this project is therefore to quantify muscular loads during different patient transfers. By following Danish nurses during a full work day, this study seeks to obtain real-life measurements during patient transfers. The key aspect of this project is the measurement of muscle load and body positions during a number of patient transfers, with and without the use of assistive devices. These technical measurements, using surface electromyography (EMG) and accelerometers to quantify muscle activity and body movements, respectively, will allow for identification of the biomechanical characteristics of various patient transfers. As preparatory work to ensure high quality data collection, the most common types of patient transfers in Danish hospitals have been identified and a pilot-study that primarily served to test the EMG and actigraphy equipment has been performed (manuscript in preparation). Furthermore, another novel aspect of this project will combine the technical measurements described above with the results from fortnightly emails received from nurses in Danish hospitals. These emails will include answers to a short questionnaire regarding the weekly intensity, frequency, and duration of patient transfers, as well as the number of back injuries (defined as a sudden and unexpected accident during a patient transfer) and the intensity of LBP (Visual Analogue Scale 0-10).

The combination of technical measurements (EMG and actigraphy) and the prospective aspect of this project will allow for the development of a detailed exposure profile [25]. Therefore, by combining the individual strengths of technical measurements and epidemiological research methods, this project seeks to answer important questions regarding the implications of utilizing technical equipment/assistive devices in Danish hospitals, and to improve electronic health (eHealth) practices among Danish nurses.

## Methods

### Study Design

**Figure 1:**
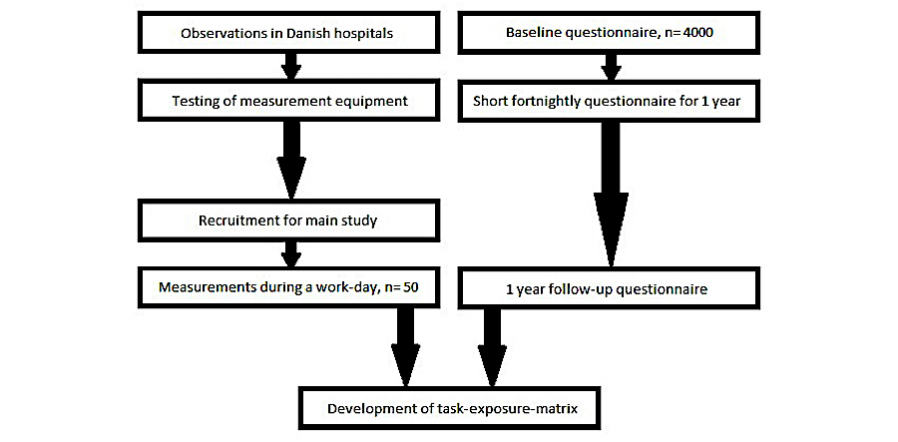
Overview of study design.

This study follows a cross-sectional (technical measurements) design as well as a prospective cohort design (questionnaires and fortnightly electronic follow-up). The technical measurements will be performed on full-time nurses from hospitals in Denmark. Therefore, by following nurses during a work day, the measurements will reflect real-life patient transfer scenarios with and without the use of appropriate assistive devices. Data collection is expected to take place from autumn 2017 to spring 2018, and baseline questionnaires were sent out in March 2017. Figure 1 shows an overview of the study design.

### Ethics

Referring to the Helsinki Declaration, all participants will be informed about the content of the study before providing written informed consent. This information will be given both written and verbally. The study (ie, the use of technical measurements) is approved by the Danish National Committee on Biomedical Research Ethics (the local ethical committee of Frederiksberg and Copenhagen; H-3-2010-062) and the Danish Data Protection Agency (j.nr. 2015-41-4232).

### Study Population and Recruitment

Approximately 50 female nurses will be recruited from Danish hospitals. To achieve a sufficient number of measurements of patient transfers during each individual work day, prospects will be approached by the lead nurse who knows the schedules. The generalizability of this study will therefore only apply to female nurses, as this constitutes a homogenous group and represents the vast majority of health care personnel in Denmark. Inclusion criteria will be an expected number of 10 or more patient transfers during the work day. Exclusion criteria are life-threatening diseases, pregnancy, and hypertension. The two former criteria will be evaluated by asking the nurse prior to the day of testing, whereas the latter will be measured on the day and is defined as readings >160/100 mmHg.

### Electromyography and Actigraphy

Each participant will be equipped with wireless surface EMG equipment (Noraxon, Arizona, USA), allowing for continuous measurements. Before electrode placement, the skin will be prepped and cleaned. EMG signals will be recorded using a bipolar EMG configuration (Blue Sensor N-00-S, Ambu A/S, Ballerup, Denmark) with an interelectrode distance of 2 centimeters [26-28]. The electrodes will be connected directly to wireless probes that will preamplify the signal (gain 400), and transmit data in real-time to a 16-channel personal computer-interface receiver (Telemyo DTS Telemetry, Noraxon, Arizona, USA). The sampling rate will be set to 1500 Hertz with a bandwidth of 10-500 Hertz to avoid aliasing. Joint angles will be continuously measured and synchronized with live EMG recordings, using two electronic accelerometers (3D DTS 24G accelerometer, Noraxon, Arizona, USA). Previous research has found strong correlations between actual loading and normalized EMG amplitude [29], making EMG measurements a valid methodology to accurately detect differences in muscle load of the magnitude we expect to see in this project.

### Data Collection

Before the beginning of a work day, the participant will enter the room designated for equipment application and testing. The EMG electrodes will be positioned bilaterally on: (1) the upper trapezius, 2 centimeters lateral from the middistance between the C7 vertebra and the acromion; (2) on the lower trapezius, 2/3 on the line from the trigonum spinea to the eighth thoracic vertebra; (3) on erector spinae longissimus, two finger widths lateral from L1; and (4) on erector spinae iliocostalis, one finger width medial from the line from the posterior spinae iliaca superior to the lowest point of the rib, at the level of L2 [30,31]. The erector spinae muscles are of primary interest, whereas the trapezius muscles will constitute a future secondary analysis in relation to the high prevalence of neck/shoulder MSDs seen among nurses.

The two accelerometers will be positioned on the lower back (just above the sacroiliac joint) and on the upper back (just below C7 vertebra). The equipment listed will be fixated with adhesive tape (Fixomull, BSN medical GmbH, Hamburg, Germany), and the strength of the signal will be confirmed. After successful application, normalization procedures will be performed. 

These procedures will consist of: (1) maximal isometric voluntary contractions (MVICs); and (2) submaximal isometric contractions, which will be performed in the morning and again in the afternoon. We will include both procedures as the latter has recently shown greater reliability for the erector spinae muscle [32]. Two trials within each procedure will be performed for all muscles. For the erector spinae muscles, both the MVICs and the submaximal isometric contractions will be performed in the prone Biering-Soerensen test-position [33,34]. Likewise, in this position, a muscle endurance test for the erector spinae muscles will be performed in the afternoon. For the upper trapezius, both normalization procedures will be performed in a standing position with 90-degree arm abduction, whereas the MVICs for the lower trapezius muscles will be performed in a prone position with 130-degree shoulder abduction [35]. Likewise, the accelerometers will be calibrated in known positions prior to fixation, as well as when positioned on the subject during (1) upright standing position, (2) bending forwards to a horizontal torso position with flexed knees, (3) maximal forward bending with straight legs, (4) arching forwards, and (5) arching sideways. The accelerometer normalizations will be performed in the morning and again in the afternoon.

Following the initialization and normalization procedures described above, the participant will start her work day. The test leader will follow the participant as she is performing various patient transfers. Given the nature of the wireless equipment, the test leader will be able to visually monitor the live EMG signals on a laptop. To ensure an adequate level of detail of all patient transfers using this setup, the test leader will observe and note the type of patient transfer and associated assistive devices, the number of nurses involved, as well as patient characteristics (age, body mass, height, and level of self-reliance). Using this methodology, it will be possible to differentiate each patient transfer into its partial lifts that together constitute the whole. This approach will provide a level of detail novel to this field and will allow for more precise measurements of exposure during patient transfers. After finishing her shift, the nurse will return to the test room for end-day normalizations. By using the average value from the standardized normalizations performed before and after the work day, as well as EMG temporal and spectral changes [36], we aim to document the potential effects of fatigue on the EMG measurements.

### Outcomes

The main outcomes of this project will be the association between low back muscle activity intensity (normalized EMG and frequency of patient transfers) and body position (position and frequency), on the risk of back injury related to patient transfer and change in intensity of LBP during a one-year period.

The first article will be descriptive in terms of muscle load and body position while using different assistive devices. The following articles will apply the results of technical measurements to the cohort (n=2000) with main outcomes, which are LBP intensity and risk of back injury during patient transfers.

### Statistics

All statistical analyses will be performed using SAS statistical software for Windows (SAS Institute, Cary, NC). Poisson regression (injury) and linear regression (LBP) models will be performed using generalized estimating equations (Proc Genmod) and linear mixed models (Proc Mixed). An alpha level of 0.05 will be accepted as significant.

## Results

Data collection is scheduled to commence during the winter of 2017.

## Discussion

This is the first project to investigate the effect of using assistive devices during patient transfers via a combination of technical measurements and a prospective cohort design. Whereas previous research has either used biomechanical measurements to identify musculoskeletal loads during work or questionnaire-based designs to quantify work-related MSDs, this combination of research methodologies enables us to create exposure matrices regarding the dose-response relationship and importance of load, intensity, frequency, and type of patient transfer, in addition their individual associations with LBP back injuries related to patient transfers. In contrast to the job-exposure matrix commonly used in epidemiological studies, this exposure matrix will provide much greater detail as it assigns job exposure according to the specific tasks performed by the nurse. Furthermore, this is the first study to perform real-life measurements on nurses during a work day and will undoubtedly result in more realistic identification of potential risk factors, which can be made readily available as part of the electronic learning material currently used by nurses. Therefore, the present study has the potential to provide essential and detailed knowledge regarding the use of assistive devices during patient transfers in hospitals, and (via improved eHealth practices) to better guide the workplaces’ efforts to reduce the number of back injuries and the occurrence of MSDs among female nurses.

## Abbreviations

eHealth: electronic health
EMG: electromyography
LBP: low back pain
MSD: musculoskeletal disorder
MVIC: maximal isometric voluntary contraction
OR: odds ratio
